# Supplementary figures and images for: First-line risk stratification with machine learning models facilitates rapid triage for non-ST-elevation myocardial infarction
Source: PLOS Digit Health. 2026 Feb 23;5(2):e0001260. doi: 10.1371/journal.pdig.0001260 (PMC12928466; doi:10.1371/journal.pdig.0001260)

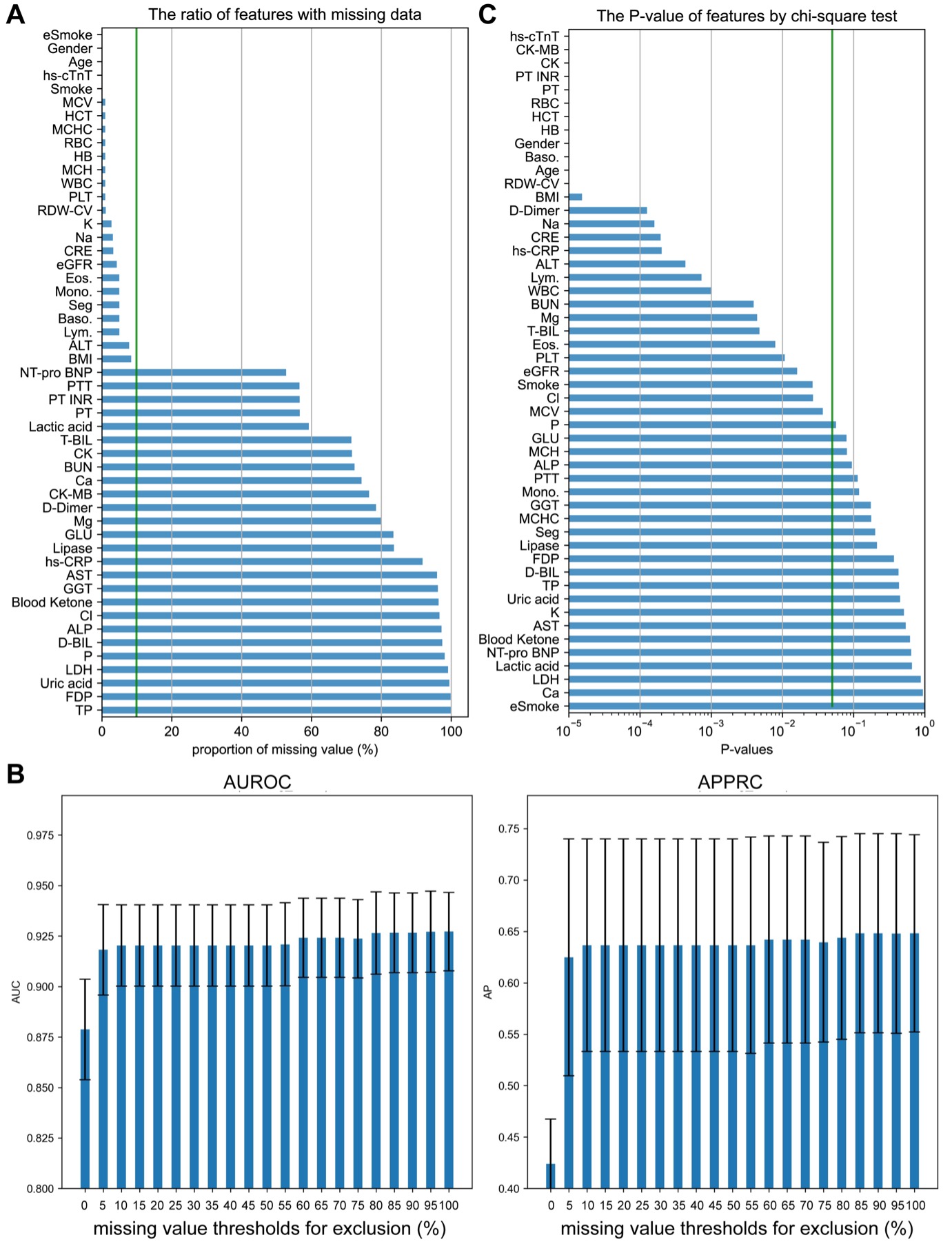

Supplement: S1 Fig — (A) The percentage of missing values for each laboratory blood test. The green line indicates the exclusion cutoff where tests with missing values greater than 10% were excluded. (B) Area under the receiver operating characteristic curve (AUROC) (left panel) and average precision of the precision-recall curve (APPRC) (right panel) of models trained with different missing value thresholds. If a feature had missing value proportion > threshold, then the feature was excluded from the feature vector. Data were mean±SD from cross-validation. (C) Features were subjected to quantile binning and p-values were calculated by chi-square test. Features with p-values > 0.05 (green line) were excluded from the feature vector. (JPG) [file pdig.0001260.s001.jpg]

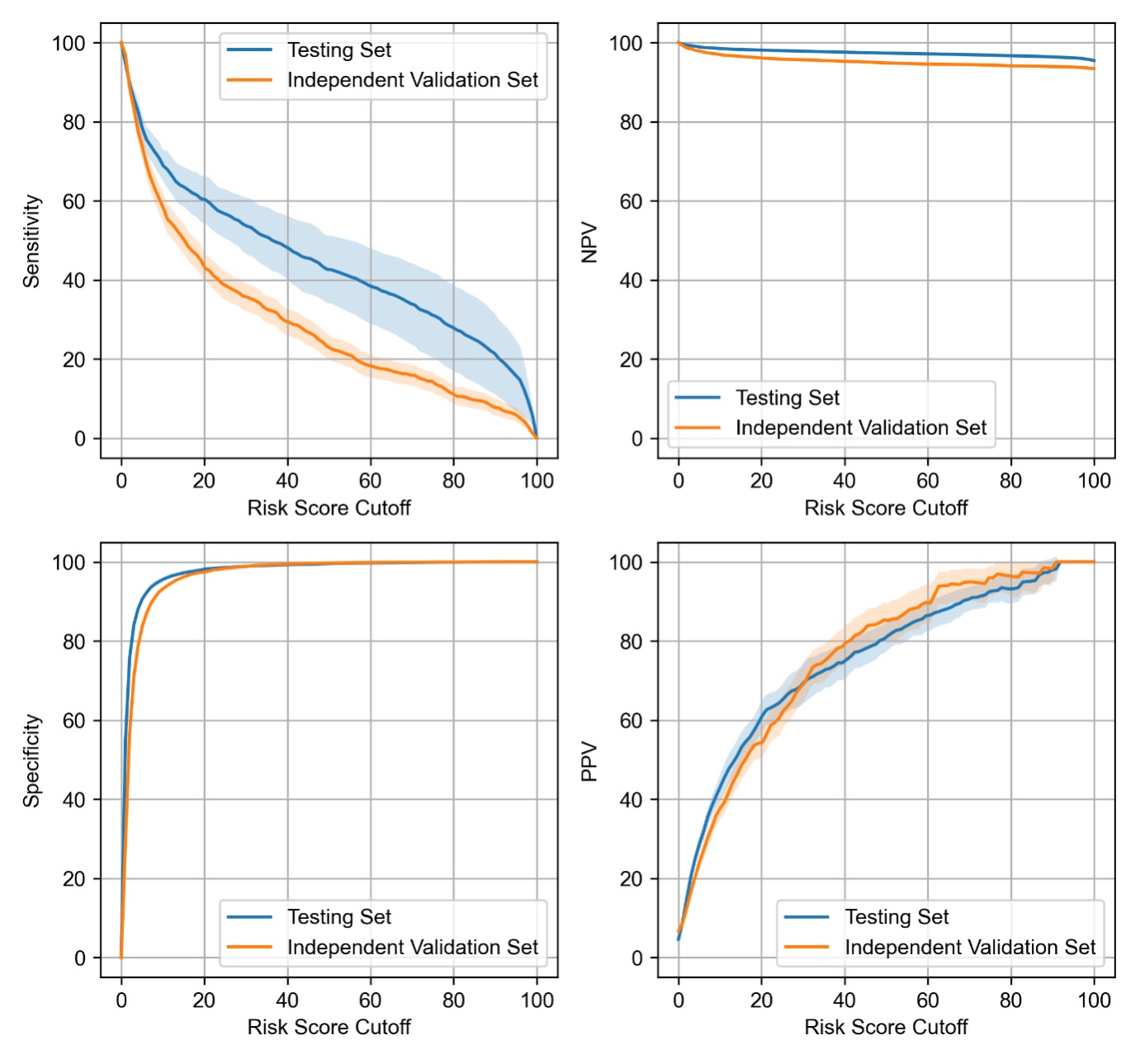

Supplement: S2 Fig — Models were tested and the probabilities generated from the XGBoost model were regarded as risk scores. Sensitivity, specificity, NPV, and PPV were estimated and plotted based on different risk score thresholds above which a patient was classified as positive and vice versa. NPV, negative predictive values; PPV, positive predictive values. Solid lines represent medians and color zones represent 95% CI. (JPG) [file pdig.0001260.s002.jpg]

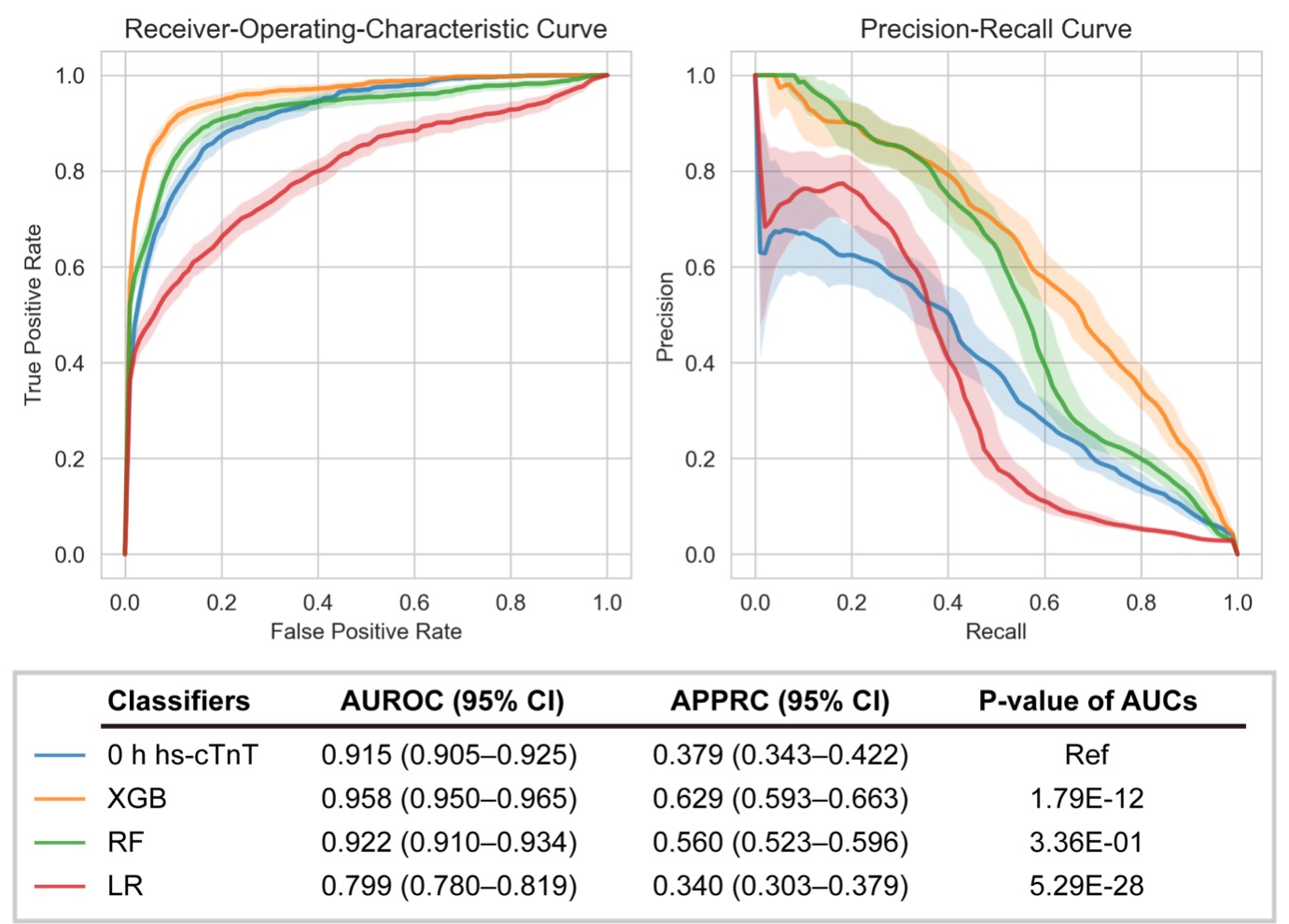

Supplement: S3 Fig — Comparison of the area under receiver-operating-curves (AUROC) (upper left) and average precision of precision-recall curves (APPRC) (upper right) generated from the patients tested only once for hs-cTnT for the XGBoost (XGB) model, random forest (RF) model, logistic regression (LR) model, and model using 0 h hs-cTnT alone as an input feature. Solid lines represent medians and color zones represent 95% CI. (JPG) [file pdig.0001260.s003.jpg]

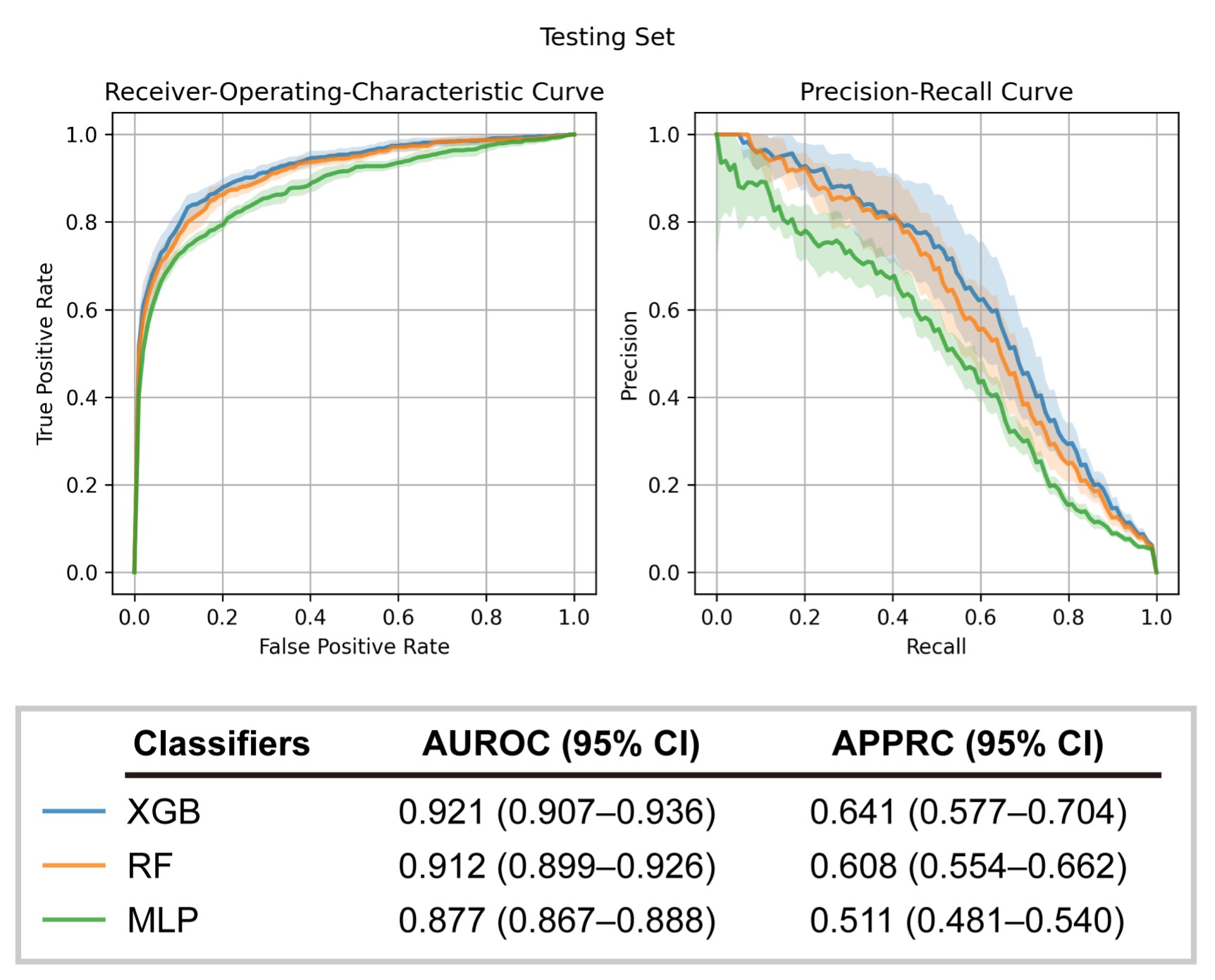

Supplement: S4 Fig — Comparison of the area under receiver-operating-curves (AUROC) (upper left) and average precision of precision-recall curves (APPRC) (upper right) generated from the testing set for the XGBoost (XGB) model, random forest (RF) model, and multilayer perceptron (MLP) architecture. Solid lines represent medians, and color zones represent 95% CI. (JPG) [file pdig.0001260.s004.jpg]
