# Supplementary material for: First-line risk stratification with machine learning models facilitates rapid triage for non-ST-elevation myocardial infarction
Source: PLOS Digit Health. 2026 Feb 23;5(2):e0001260. doi: 10.1371/journal.pdig.0001260 (PMC12928466; doi:10.1371/journal.pdig.0001260)
Supplement: S1 Table — (DOCX) [file pdig.0001260.s005.docx]

**S1 Table. Full name of the abbreviation of laboratory tests and units.**

NA, not applicable.

| **Abbreviation** | **Full Name** | **Unit** |
| --- | --- | --- |
| **ALP** | Alkaline Phosphatase | U/L |
| **ALT** | Alanine Aminotransferase | U/L |
| **AST** | Aspartate Aminotransferase | U/L |
| **BUN** | Blood Urea Nitrogen | mg/dL |
| **Baso.** | Basophil | % |
| **Blood Ketone** | Blood Ketone | mmol/L |
| **CK** | Creatine Kinase | U/L |
| **CK-MB** | Creatine Kinase MB | ng/mL |
| **CRE** | Creatinine | mg/dL |
| **Ca** | Calcium | mmol/L |
| **Cl** | Chloride | mmol/L |
| **D-BIL** | Bilirubin, Direct | mg/dL |
| **D-Dimer** | D-Dimer | μg/mL FEU |
| **Eos.** | Eosinophil | % |
| **FDP** | Fibrinogen Degradation Products | μg/mL |
| **GGT** | Gamma-Glutamyl Transferase | U/L |
| **GLU** | Glucose | mg/dL |
| **HB** | Hemoglobin | g/dL |
| **HCT** | Hematocrit | % |
| **K** | Potassium | mmol/L |
| **LDH** | Lactate Dehydrogenase | U/L |
| **Lactic acid** | Lactic Acid | mmol/L |
| **Lipase** | Lipase | U/L |
| **Lym.** | Lymphocyte | % |
| **MCH** | Mean Corpuscular Hemoglobin | pg |
| **MCHC** | Mean Corpuscular Hemoglobin Concentration | g/dL |
| **MCV** | Mean Corpuscular Volume | fL |
| **Mg** | Magnesium | mmol/L |
| **Mono.** | Monocyte | % |
| **NT-pro BNP** | N-terminal pro B-type natriuretic peptide | pg/mL |
| **Na** | Sodium | mmol/L |
| **P** | Phosphate | mg/dL |
| **PLT** | Platelet | K/μL |
| **PT** | Prothrombin Time | sec |
| **PT INR** | Prothrombin Time and International Normalized Ratio | NA |
| **PTT** | Partial Thromboplastin Time | sec |
| **RBC** | Red Blood Cell | M/μL |
| **RDW-CV** | Red Cell Volume Distribution Width | % |
| **Seg** | Segmented Neutrophil | % |
| **T-BIL** | Total Bilirubin | mg/dL |
| **TP** | Total Protein | g/dL |
| **Uric acid** | Uric Acid | mg/dL |
| **WBC** | White Blood Cell | K/μL |
| **eGFR** | Estimated Glomerular Filtration Rate | mL/min/1.73 m^2 |
| **hs-CRP** | High-Sensitivity C-Reactive Protein | mg/dL |
| **hs-cTnT** | High-Sensitive Cardiac Troponin T | ng/L |
