# Supplementary material for: First-line risk stratification with machine learning models facilitates rapid triage for non-ST-elevation myocardial infarction
Source: PLOS Digit Health. 2026 Feb 23;5(2):e0001260. doi: 10.1371/journal.pdig.0001260 (PMC12928466; doi:10.1371/journal.pdig.0001260)
Supplement: S2 Table — (DOCX) [file pdig.0001260.s006.docx]

**S2 Table. The setting of tuned hyper-parameters used to train machine learning models.**

|  | **Random forest** | **Logistic regression** | **XGBoost** |
| --- | --- | --- | --- |
| **n_estimators** | 700 |  | 600 |
| **max_features** | log2 |  |  |
| **criterion** | entropy |  |  |
| **min_samples_leaf** | 8 |  |  |
| **min_samples_split** | 8 |  |  |
| **min_weight_fraction_leaf** | 0 |  |  |
| **max_depth** | 7 |  | 6 |
| **max_leaf_nodes** | None |  |  |
| **max_samples** | 0.8 |  |  |
| **min_impurity_decrease** | 0 |  |  |
| **class_weight** | balanced_subsample | balanced |  |
| **penalty** |  | l2 |  |
| **fit_intercept** |  | TRUE |  |
| **max_iter** |  | 250 |  |
| **C** |  | 950 |  |
| **tol** |  | 1.13E-07 |  |
| **subsample** |  |  | 0.7 |
| **learning_rate** |  |  | 0.016662 |
| **alpha** |  |  | 4.91E-05 |
| **lambda** |  |  | 0.000343 |
| **gamma** |  |  | 0.218536 |
| **min_child_weight** |  |  | 8 |
| **grow_policy** |  |  | lossguide |
| **colsample_bytree** |  |  | 0.3 |
| **scale_pos_weight** |  |  | 1 |
