# Supplementary material for: First-line risk stratification with machine learning models facilitates rapid triage for non-ST-elevation myocardial infarction
Source: PLOS Digit Health. 2026 Feb 23;5(2):e0001260. doi: 10.1371/journal.pdig.0001260 (PMC12928466; doi:10.1371/journal.pdig.0001260)
Supplement: S3 Table — (DOCX) [file pdig.0001260.s007.docx]

**S3 Table. Summary of the statistics and P-values of routine laboratory tests measured from the first blood draw.**

Data are median (IQR).

| **Medical Center** | **National Taiwan University Hospital** | | | | **Hsin-Chu Branch of National Taiwan University Hospital** | | | |
| --- | --- | --- | --- | --- | --- | --- | --- | --- |
| **Blood Test** | **Total Median (25%–75% quantile)** | **Case Median (25%–75% quantile)** | **Control Median (25%–75% quantile)** | **P-value** | **Total Median (25%–75% quantile)** | **Case Median (25%–75% quantile)** | **Control Median (25%–75% quantile)** | **P-value** |
| **hs-cTnT** | 27.4 (14.4, 66.4) | 146.8 (39.5, 553.5) | 26.2 (14.1, 60.9) | 7.67E-185 | 26.4 (13.3, 62.2) | 72.8 (24.5, 282.6) | 25.1 (12.4, 57.2) | 6.47E-133 |
| **CK-MB** | 2.9 (1.6, 5.9) | 11.6 (4.2, 31.1) | 2.6 (1.5, 4.7) | 9.04E-70 | 2.9 (1.7, 5.6) | 7.3 (3.0, 16.9) | 2.7 (1.6, 4.6) | 7.13E-42 |
| **HB** | 12.5 (10.4, 14.2) | 13.6 (11.3, 15.1) | 12.4 (10.3, 14.1) | 1.62E-16 | 12.6 (10.5, 14.4) | 13.6 (11.1, 15.1) | 12.6 (10.5, 14.3) | 1.76E-10 |
| **HCT** | 38.0 (32.0, 42.9) | 41.3 (35.0, 45.0) | 37.9 (31.9, 42.8) | 3.22E-15 | 37.8 (32.1, 42.7) | 40.6 (34.3, 44.7) | 37.7 (32.0, 42.6) | 7.19E-11 |
| **RBC** | 4.2 (3.5, 4.8) | 4.6 (3.8, 5.1) | 4.2 (3.5, 4.8) | 3.23E-14 | 4.2 (3.6, 4.8) | 4.5 (3.9, 5.0) | 4.2 (3.5, 4.8) | 7.24E-11 |
| **PTT** | 27.6 (25.3, 30.7) | 27.3 (25.2, 30.4) | 27.6 (25.3, 30.8) | 4.04E-13 | 27.4 (25.1, 30.3) | 27.4 (25.3, 31.1) | 27.4 (25.1, 30.2) | 5.79E-19 |
| **RDW-CV** | 13.7 (12.9, 15.4) | 13.2 (12.7, 14.5) | 13.8 (12.9, 15.4) | 1.96E-09 | 13.7 (12.9, 15.2) | 13.4 (12.8, 14.7) | 13.7 (12.9, 15.2) | 2.23E-03 |
| **Lym.** | 16.9 (9.3, 26.0) | 20.1 (12.4, 28.2) | 16.7 (9.2, 26.0) | 4.83E-07 | 18.0 (10.4, 27.0) | 20.4 (12.5, 29.6) | 17.9 (10.2, 26.7) | 1.70E-07 |
| **PT** | 11.0 (10.4, 12.0) | 10.6 (10.3, 11.0) | 11.0 (10.4, 12.1) | 3.12E-05 | 10.6 (10.1, 11.4) | 10.4 (9.9, 10.9) | 10.6 (10.2, 11.5) | 2.18E-04 |
| **PT INR** | 1.0 (1.0, 1.1) | 1.0 (1.0, 1.0) | 1.0 (1.0, 1.1) | 3.18E-05 | 1.0 (1.0, 1.1) | 1.0 (1.0, 1.1) | 1.0 (1.0, 1.1) | 1.36E-03 |
| **hs-CRP** | 2.4 (0.4, 9.9) | 0.9 (0.2, 3.6) | 2.6 (0.4, 10.3) | 4.31E-04 | 1.3 (0.2, 5.4) | 0.7 (0.2, 3.2) | 1.3 (0.2, 5.5) | 2.11E-02 |
| **Na** | 135.0 (132.0, 138.0) | 136.0 (133.0, 138.0) | 135.0 (132.0, 138.0) | 4.70E-04 | 136.0 (133.0, 139.0) | 137.0 (134.0, 139.0) | 136.0 (133.0, 139.0) | 1.02E-02 |
| **BUN** | 34.8 (20.6, 62.3) | 26.9 (17.1, 47.2) | 35.1 (20.8, 62.8) | 8.64E-04 | 31.0 (19.9, 57.0) | 26.0 (17.0, 48.0) | 31.0 (20.0, 58.0) | 4.16E-02 |
| **MCHC** | 32.9 (31.9, 33.8) | 33.0 (32.1, 33.9) | 32.9 (31.9, 33.8) | 1.13E-03 | 33.3 (32.4, 34.3) | 33.4 (32.4, 34.4) | 33.3 (32.4, 34.3) | 2.08E-01 |
| **P** | 4.1 (3.2, 6.0) | 3.5 (3.1, 4.2) | 4.3 (3.2, 6.2) | 7.27E-03 | 3.8 (2.7, 5.1) | 5.2 (4.2, 6.2) | 3.8 (2.5, 5.0) | 6.20E-01 |
| **Baso.** | 0.4 (0.2, 0.6) | 0.4 (0.3, 0.6) | 0.4 (0.2, 0.6) | 8.43E-03 | 0.4 (0.2, 0.6) | 0.4 (0.2, 0.6) | 0.4 (0.2, 0.6) | 4.41E-01 |
| **Mg** | 0.9 (0.8, 1.0) | 0.9 (0.8, 1.0) | 0.9 (0.8, 1.0) | 1.03E-02 | 2.0 (1.8, 2.2) | 2.0 (1.7, 2.3) | 2.0 (1.8, 2.2) | 4.64E-01 |
| **D-Dimer** | 0.8 (0.3, 2.1) | 0.5 (0.3, 1.0) | 0.8 (0.3, 2.2) | 1.54E-02 | 0.7 (0.3, 1.8) | 0.5 (0.3, 1.3) | 0.7 (0.3, 1.8) | 1.38E-01 |
| **T-BIL** | 0.7 (0.5, 1.1) | 0.6 (0.5, 0.8) | 0.7 (0.5, 1.1) | 2.52E-02 | 0.7 (0.5, 1.1) | 0.6 (0.4, 0.9) | 0.7 (0.5, 1.1) | 1.61E-01 |
| **ALP** | 90.0 (60.0, 169.0) | 74.0 (51.0, 86.0) | 93.0 (60.0, 177.0) | 2.75E-02 | 81.5 (64.2, 122.8) | 68.0 (61.5, 85.5) | 84.0 (64.5, 125.0) | 5.92E-01 |
| **Seg** | 73.3 (63.2, 82.8) | 71.4 (61.5, 80.5) | 73.3 (63.3, 82.8) | 4.50E-02 | 69.5 (59.7, 80.1) | 65.7 (54.8, 78.2) | 69.9 (60.2, 80.4) | 2.71E-03 |
| **Cl** | 100.0 (95.0, 106.0) | 103.5 (99.0, 106.8) | 100.0 (95.0, 106.0) | 5.83E-02 | 98.0 (93.0, 103.0) | 97.0 (93.2, 103.8) | 98.0 (93.0, 103.0) | 8.40E-01 |
| **GGT** | 51.0 (20.0, 152.5) | 37.0 (19.0, 63.0) | 53.0 (20.2, 162.2) | 6.20E-02 | 29.5 (20.5, 136.2) | 91.0 (56.0, 107.0) | 27.0 (19.0, 176.0) | 6.52E-01 |
| **K** | 4.1 (3.7, 4.6) | 4.0 (3.7, 4.5) | 4.1 (3.7, 4.6) | 8.65E-02 | 3.9 (3.6, 4.4) | 3.9 (3.6, 4.4) | 4.0 (3.6, 4.4) | 5.51E-01 |
| **Eos.** | 1.0 (0.1, 2.1) | 1.1 (0.4, 2.5) | 1.0 (0.1, 2.1) | 1.00E-01 | 1.1 (0.3, 2.4) | 1.3 (0.5, 2.6) | 1.1 (0.3, 2.4) | 2.79E-02 |
| **D-BIL** | 0.7 (0.2, 2.5) | 0.4 (0.3, 0.7) | 0.7 (0.2, 2.5) | 1.26E-01 | 0.3 (0.1, 1.1) | 0.2 (0.1, 0.3) | 0.3 (0.1, 1.2) | 3.12E-01 |
| **NT-pro BNP** | 1482.0 (328.1, 5736.0) | 1492.0 (346.1, 7026.0) | 1481.5 (327.2, 5711.2) | 1.47E-01 | 1318.5 (269.2, 5782.8) | 2456.5 (356.1, 10770.0) | 1231.5 (262.1, 5457.0) | 1.34E-05 |
| **Mono.** | 5.7 (4.2, 7.1) | 5.6 (4.4, 6.8) | 5.7 (4.2, 7.1) | 1.53E-01 | 5.8 (4.5, 7.3) | 5.5 (4.4, 6.8) | 5.8 (4.5, 7.3) | 3.55E-02 |
| **eGFR** | 62.4 (34.2, 87.0) | 67.7 (43.3, 88.0) | 62.1 (33.9, 87.0) | 1.56E-01 | 56.6 (32.5, 81.8) | 63.1 (37.0, 84.4) | 55.9 (32.1, 81.3) | 4.36E-01 |
| **Blood Ketone** | 0.4 (0.2, 1.1) | 0.3 (0.3, 0.6) | 0.4 (0.2, 1.1) | 2.15E-01 | 0.3 (0.2, 0.8) | 0.6 (0.2, 0.8) | 0.3 (0.2, 0.9) | 6.36E-01 |
| **Lactic acid** | 2.3 (1.5, 4.0) | 2.1 (1.5, 3.3) | 2.3 (1.5, 4.0) | 2.22E-01 | 1.9 (1.3, 3.1) | 2.2 (1.5, 3.2) | 1.9 (1.3, 3.1) | 1.00E-01 |
| **MCV** | 91.3 (87.3, 95.2) | 91.0 (87.5, 94.2) | 91.3 (87.3, 95.3) | 2.66E-01 | 90.3 (86.2, 94.3) | 90.1 (86.6, 93.7) | 90.3 (86.2, 94.3) | 2.73E-01 |
| **CK** | 98.0 (59.0, 174.0) | 176.0 (95.0, 352.8) | 93.0 (56.0, 158.0) | 3.65E-01 | 103.0 (61.8, 204.5) | 136.0 (79.0, 246.0) | 100.0 (59.0, 190.5) | 8.27E-01 |
| **PLT** | 211.0 (163.0, 267.0) | 215.0 (178.0, 262.0) | 211.0 (162.0, 267.0) | 3.85E-01 | 224.0 (177.0, 281.0) | 233.0 (180.0, 286.0) | 223.0 (176.8, 279.2) | 8.92E-01 |
| **ALT** | 18.0 (12.0, 29.0) | 20.0 (14.0, 30.0) | 18.0 (12.0, 29.0) | 3.90E-01 | 19.0 (12.0, 30.0) | 20.0 (14.0, 30.0) | 19.0 (12.0, 30.0) | 5.44E-01 |
| **Uric acid** | 7.0 (5.2, 9.8) | 7.0 (6.1, 8.2) | 7.3 (5.2, 10.1) | 4.20E-01 | 7.2 (5.7, 8.8) | 8.8 (7.8, 9.8) | 7.2 (5.3, 8.2) | 3.97E-01 |
| **Lipase** | 28.0 (15.0, 53.0) | 25.5 (17.0, 49.5) | 28.0 (14.0, 53.0) | 4.29E-01 | 34.0 (19.0, 56.4) | 32.9 (21.0, 52.1) | 34.0 (19.0, 56.8) | 4.33E-01 |
| **Ca** | 2.3 (2.2, 2.4) | 2.3 (2.2, 2.4) | 2.3 (2.2, 2.4) | 4.90E-01 | 2.3 (2.2, 2.4) | 2.3 (2.3, 2.4) | 2.3 (2.2, 2.4) | 7.92E-01 |
| **AST** | 40.0 (22.0, 97.0) | 58.0 (27.0, 100.0) | 39.0 (22.0, 96.8) | 5.69E-01 | 28.0 (19.0, 54.0) | 32.0 (19.0, 60.0) | 28.0 (19.0, 52.0) | 2.91E-02 |
| **MCH** | 30.2 (28.7, 31.5) | 30.3 (29.0, 31.4) | 30.2 (28.7, 31.5) | 5.93E-01 | 30.3 (28.8, 31.7) | 30.4 (28.8, 31.5) | 30.3 (28.8, 31.7) | 7.12E-01 |
| **TP** | 6.9 (6.5, 7.3) | 7.0 (6.9, 7.2) | 6.9 (6.5, 7.2) | 6.49E-01 | 7.1 (6.5, 7.8) | 6.5 (6.5, 6.5) | 7.2 (6.6, 7.9) | 0.00E+00 |
| **LDH** | 426.5 (259.0, 766.5) | 410.0 (368.5, 788.5) | 427.0 (255.0, 742.0) | 7.45E-01 | 254.5 (181.2, 365.0) | 304.5 (187.5, 478.0) | 252.5 (178.0, 351.0) | 7.36E-01 |
| **GLU** | 138.0 (110.0, 191.5) | 166.0 (114.0, 224.0) | 137.0 (110.0, 189.8) | 7.59E-01 | 130.0 (107.0, 172.0) | 154.0 (123.0, 220.5) | 128.0 (107.0, 170.0) | 8.60E-10 |
| **WBC** | 8.2 (6.2, 11.2) | 8.8 (6.9, 11.5) | 8.2 (6.2, 11.2) | 7.63E-01 | 8.5 (6.5, 11.2) | 9.0 (7.1, 11.3) | 8.4 (6.4, 11.1) | 9.23E-02 |
| **CRE** | 1.1 (0.8, 1.8) | 1.1 (0.9, 1.6) | 1.1 (0.8, 1.8) | 9.49E-01 | 1.2 (0.9, 1.9) | 1.2 (0.8, 1.7) | 1.2 (0.9, 1.9) | 7.92E-01 |
