# Supplementary material for: First-line risk stratification with machine learning models facilitates rapid triage for non-ST-elevation myocardial infarction
Source: PLOS Digit Health. 2026 Feb 23;5(2):e0001260. doi: 10.1371/journal.pdig.0001260 (PMC12928466; doi:10.1371/journal.pdig.0001260)
Supplement: S4 Table — (DOCX) [file pdig.0001260.s008.docx]

**S4 Table.** **Performance of models trained with or without CKs/PTs features.**

Machine learning models were trained with routine laboratory tests containing CKs/PTs or not and model performance was tested on the testing set. CKs represent CK (Creatine Kinase) and CK-MB (Creatine Kinase MB). PTs represent PT (Prothrombin Time) and PT INR (Prothrombin Time International Normalized Ratio).

| **Classifier** | **CKs/PTs** | **AUROC (95% CI)** | **APPRC (95% CI)** | **P-value of AUCs** |
| --- | --- | --- | --- | --- |
| **0 h hs-cTnT** | **−** | 0.775 (0.773–0.778) | 0.242 (0.216–0.268) | Ref |
| **XGBoost** | **+** | 0.921 (0.907–0.936) | 0.641 (0.577–0.704) | 1.15E-05 |
| **XGBoost** | **−** | 0.877 (0.856–0.898) | 0.531 (0.446–0.617) | 1.72E-03 |
| **Random forest** | **+** | 0.912 (0.899–0.926) | 0.608 (0.554–0.662) | 1.24E-04 |
| **Random forest** | **−** | 0.870 (0.854–0.885) | 0.509 (0.426–0.591) | 1.59E-02 |
| **Logistic regression** | **+** | 0.846 (0.834–0.858) | 0.389 (0.362–0.417) | 1.79E-01 |
| **Logistic regression** | **−** | 0.806 (0.791–0.821) | 0.270 (0.246–0.295) | 3.13E-01 |
