# Supplementary material for: First-line risk stratification with machine learning models facilitates rapid triage for non-ST-elevation myocardial infarction
Source: PLOS Digit Health. 2026 Feb 23;5(2):e0001260. doi: 10.1371/journal.pdig.0001260 (PMC12928466; doi:10.1371/journal.pdig.0001260)
Supplement: S5 Table — (DOCX) [file pdig.0001260.s009.docx]

**S5 Table. Model performance on the testing set based on different risk score cutoffs derived to meet predefined diagnostic criteria.**

Sensitivity and negative predictive value (NPV) thresholds categorize the population into low-risk and non-low-risk groups, without establishing a high-risk group. Similarly, specificity and positive predictive value (PPV) thresholds categorize the population into high-risk and non-high-risk groups, without establishing a low-risk group. Data are median (95% CI).

| **Statistical**  **thresholds** | **Risk score**  **thresholds** | **Sensitive, %** | **NPV, %** | **Specificity, %** | **PPV, %** | **Proportion**  **Low Risk, %** | **Proportion**  **High Risk, %** |
| --- | --- | --- | --- | --- | --- | --- | --- |
| **Sensitivity**  **≥ 95.0%** | 1.2 (0.9–1.5) | 95.7 (95.7–95.7) | 99.6 (99.5–99.7) | 56.2 (47.4–65.0) | 10.3 (8.3–12.2) | 53.8 (45.4–62.3) | … |
| **NPV**  **≥ 99.5%** | 1.8 (1.2–2.4) | 93.2 (91.5–94.9) | 99.5 (99.4–99.6) | 64.4 (48.7–80.0) | 14.0 (10.5–17.6) | 61.7 (46.7–76.8) | … |
| **Specificity**  **≥ 99.0%** | 33.4 (30.3–36.5) | 51.7 (43.9–59.6) | 97.7 (97.4–98.1) | 99.0 (99.0–99.0) | 71.0 (67.9–74.2) | … | 3.3 (2.9–3.7) |
| **PPV**  **≥ 75.0%** | 38.5 (32.5–44.4) | 49.4 (39.8–59.1) | 97.6 (97.2–98.1) | 99.2 (99.1–99.4) | 75.5 (75.2–75.7) | … | 3.0 (2.4–3.6) |
