# Supplementary material for: First-line risk stratification with machine learning models facilitates rapid triage for non-ST-elevation myocardial infarction
Source: PLOS Digit Health. 2026 Feb 23;5(2):e0001260. doi: 10.1371/journal.pdig.0001260 (PMC12928466; doi:10.1371/journal.pdig.0001260)
Supplement: Attachment — Submitted filename: Response to Reviewers.docx [file pdig.0001260.s010.docx]

Dear Editors of *PLOS Digital Health* Prof. Lamichhane and Prof. Celi

We are profoundly grateful for the opportunity to revise and resubmit our manuscript entitled “**First-line risk stratification with machine learning models facilitates rapid triage for non-ST-elevation myocardial infarction**” (PDIG-D-25-00558) for possible publication in *PLOS Digital Health*.

We sincerely appreciate the Editors’ and Reviewers’ thoughtful and highly constructive comments. These insightful suggestions – particularly those concerning feature selection approaches, exclusion criteria, missing-data handing, addressing low-prevalence issues, benchmarking with alternative algorithms, and the clinical integration of the ML + ESC 0/1 h framework – have been invaluable in strengthening both the scientific rigor and translational relevance of our work.

In preparing this revised manuscript, we have carefully addressed every comments. To fully respond to reviewers’ requests, we conducted additional data analysis and model evaluations, including external validation procedures, and performance comparisons with neural network models and gradient boosting in MI^3^. These additional analyses required substantial time but significantly improved the robustness of the findings.

Following the reviewers’ recommendations, we have implemented major revisions to several sections, including Methods, Results, and Discussion, to clarify study design, justify methodological choices, and more effectively contextualize the clinical implications. We also performed comprehensive language refinement and conceptual clarification throughout the manuscript to improve precision, coherence, and readability. For transparency and clarity in the review process, we have provided the following documents:

1. A detailed, point-by-point response to each reviewer’s comment, clearly explaining the specific changes made and where they can be found in the manuscript.
2. A tracked-changes version of the manuscript to facilitate the review of all modifications.
3. A clean revised version for ease of reading.
4. We also now uploaded high-resolution standalone figure files, and all figure legends and tables are consolidated at the end of the revised manuscript, as per journal guidelines.

We genuinely value this opportunity to refine our work, and we are truly grateful for the Editors’ and Reviewers’ dedication and time spent evaluating our study. We firmly believe that the revised manuscript not only better reflects the scientific rigor expected by *PLOS Digital Health* but also provides meaningful and clinically actionable insights for improving early decision-making and patient outcomes in acute cardiac care.

We sincerely hope that our efforts meet your expectations and that the revised manuscript will be favorably considered for publication. Thank you very much for your kind attention and continued guidance.

Sincerely yours,

Kang-Yi Su Ph.D.
Professor
Department of Clinical Laboratory Sciences and Medical Biotechnology, College of Medicine, National Taiwan University
No.1, Sec 1., Jen-Ai Road, Taipei 10055, Taiwan, R.O.C
Tel: 886-2-23123456 ext. 266910

Dear Dr. Su,

Thank you for submitting your manuscript to PLOS Digital Health. After careful consideration, we feel that it has merit but does not fully meet PLOS Digital Health's publication criteria as it currently stands. Therefore, we invite you to submit a revised version of the manuscript that addresses the points raised during the review process.

 Please submit your revised manuscript within 60 days Dec 26 2025 11:59PM. If you will need more time than this to complete your revisions, please reply to this message or contact the journal office at [digitalhealth@plos.org](mailto:digitalhealth@plos.org). When you're ready to submit your revision, log on to <https://www.editorialmanager.com/pdig/> and select the 'Submissions Needing Revision' folder to locate your manuscript file.

Please include the following items when submitting your revised manuscript:

 * A rebuttal letter that responds to each point raised by the editor and reviewer(s). You should upload this letter as a separate file labeled 'Response to Reviewers'. This file does not need to include responses to any formatting updates and technical items listed in the 'Journal Requirements' section below.

* A marked-up copy of your manuscript that highlights changes made to the original version. You should upload this as a separate file labeled 'Revised Manuscript with Track Changes'.

 * An unmarked version of your revised paper without tracked changes. You should upload this as a separate file labeled 'Manuscript'.

If you would like to make changes to your financial disclosure, competing interests statement, or data availability statement, please make these updates within the submission form at the time of resubmission. Guidelines for resubmitting your figure files are available below the reviewer comments at the end of this letter.

 We look forward to receiving your revised manuscript.

Kind regards,

Bishal Lamichhane

Guest Editor

 PLOS Digital Health

Leo Anthony Celi

Editor-in-Chief

PLOS Digital Health

orcid.org/0000-0001-6712-6626

**Additional Editor Comments (if provided):**

 This paper addresses a clinically relevant problem of NSTEMI detection using machine learning by leveraging routinely collected clinical and demographic data. The paper was reviewed by three experts in the field. All reviewers are positive about the contribution of the work, highlighting the clinical relevance and independent validation as a strength. However, there are some outstanding concerns and clarifications required. Specially, the paper should better clarify feature selection approach, exclusion criteria, address the evaluation metrics in light of low prevalences, and extend the benchmarkings. Please refer to the reviewers' comments for details.

**Response:**

We sincerely appreciate the reviewers’ recognition of the clinical relevance and contribution of our work. We are truly encouraged that our study was viewed positively regarding its independent validation and potential impact. Beyond introducing novel insights to the readers of *PLOS Digital Health*, our ultimate goal is to facilitate practical implementation in emergency departments to address currently unmet clinical needs. We hope our approach can help bridge the gap between intelligent digital health technologies and real-world clinical decision-making.

In this context, we fully acknowledge and agree with the valuable comments provided by the reviewers concerning the feature selection approach, exclusion criteria, evaluation metrics in light of low prevalence, and the extension of benchmarking analyses. We have carefully revised the manuscript to clarify and strengthen these aspects, with the sincere hope that our responses and improvements will meet the reviewers’ expectations and further enhance the scientific value of our study.

**Reviewers' Comments:**

**Reviewer #1:**

This manuscript presents a well-executed retrospective study demonstrating the potential of a ML model to enhance NSTEMI risk stratification and triage of patients with suspected ACS at the Emergency Department (ED). The core strength lies in leveraging readily available demographic and routine laboratory data from the initial blood draw alongside high-sensitivity cardiac troponin T (hs-cTnT), effectively accelerating the diagnostic process compared to relying on serial hs-cTn testing alone. Some aspects should be considered:
The overall NSTEMI prevalence is low in the training/testing set (690 cases out of 15,096, ∼4.5%). This inherently inflates the NPV and the AUC. The authors should discuss the expected performance in clinical settings with potentially higher NSTEMI prevalence.

**Response:**

We sincerely thank the reviewer for raising this highly practical and important point. Indeed, a low prevalence can reduce the PPV while inflating both the NPV and the AUC. In real-world settings, however, the proportion of patients presenting to the emergency department with chest pain who are ultimately diagnosed with AMI is approximately 5%, among whom around 60% are classified as NSTEMI. Thus, the overall prevalence of NSTEMI is roughly 3% [1, 2]. This relatively low prevalence highlights an unmet clinical need – accurate and efficient rule-out of non-AMI patients in the emergency setting.

In our study, the overall NSTEMI prevalence was approximately 4.5%, which is comparable to real-world observations. We acknowledge that the low prevalence may lead to overestimation of the NPV and AUC. In accordance with the reviewer’s valuable suggestion, we have added a statement in the Discussion section to emphasize this limitation and to note that future model recalibration may be necessary in regions where NSTEMI prevalence differs substantially (line 413-416).

*Line 413-416*

First, the observed low prevalence of NSTEMI (~4.5%) is consistent with real-world data [1, 2], but this value may have slightly overestimated the NPV and AUC. Recalibration of the model may be necessary when applying it to populations with significantly different disease prevalence.

The achieved NPV of 98.8% is slightly lower than published results for the ESC 0/1h algorithm even so the NSTEMI prevalence was much higher in the studies evaluating the 0/1h algorithm. While the rule-out capability (NPV 98.8%, excluding 48.3% of patients) is acceptable, the rule-in capability (PPV 78.1%, ruling in only 2.6% of patients) is modest in terms of coverage. The major benefit is thus in ruling out NSTEMI and safely accelerating the discharge or observation path for low-risk patients, rather than definitively ruling in the diagnosis for a large cohort. This should be highlighted in the conclusions.

**Response:**

We sincerely thank the reviewer for this insightful and highly practical comment.

We fully agree with the reviewer that, according to the ESC 0/1 h algorithm, an NPV of 98.8% appears slightly lower than what has been reported in previous validation studies. However, it is important to note that the clinical contexts of these two algorithms (0/1 h algorithm and our machine learning algorithm) differ. The ESC 0/1 h algorithm derives its NPV from a combination of both the initial and the 1-hour follow-up blood samples, and nearly one-third of patients cannot be definitively diagnosed after the first sample alone. In contrast, our proposed algorithm calculates the NPV based solely on the initial (0h) blood test, yet still achieves an NPV of 98.8%. When combined with the current ESC 0/1 h algorithm, the NPV increases to 100% (Fig 3B).

Therefore, we would like to emphasize that the major benefit of our model, as correctly highlighted by the reviewer, lies in its potential for “ruling our NSTEMI and safely accelerating the discharge or observation pathway for low-risk patients.” The goal of our study is not to replace the established algorithm, but rather to complement it and enhance its clinical utility. We have added a few clarifying sentences in the revised manuscript to reflect this explanation (line 393-397).

*Line 393-397*

Using only the 0 h sample, our model achieved an NPV of 98.8%, which increased to 100% when integrated with the ESC 0/1 h protocol (Fig 3B), underscoring its clinical utility in safely ruling out NSTEMI and facilitating timely discharge or observation of low-risk patients. This approach may shorten ED stays and improve overall efficiency for both clinicians and patients (Fig 4).

There is potential bias due to 26,761 patients whose diagnosis did not depend on a second hs-cTnT measurement. This means the model was specifically trained on the population that is diagnostically challenging (i.e., those in the "intermediate risk" grey zone that require serial testing). This limits the model’s generalizability to other population presenting to the ED.

**Response:**

We sincerely thank the reviewer for raising this critical and insightful question. Our detailed responses are as follows:

1. **Study rationale:**

The primary aim of this study was to address an unmet clinical need – patients whose diagnosis could not be determined by the initial (0 h) hs-cTnT measurement alone and who therefore require additional 1 h or 3 h blood sampling for diagnostic confirmation. To overcome this limitation, we developed a machine learning-based algorithm designed to improve diagnostic accuracy at the time of the first blood draw.

2. **Patient selection:**

Accordingly, we excluded 26,761 patients who lacked serial hs-cTnT measurements, as most of them had extremely elevated hs-cTnT levels at the initial sampling, sufficient for immediate diagnosis. This exclusion ensure that the study population accurately reflected the intended target group and was consistent with the original research purpose.

3. **Model validation on excluded population:**

We acknowledge that this design inherently led to “a model specifically trained on diagnostically challenging patients.” To address this, we further applied the developed algorithm to the 26,761 excluded patients whose diagnosis was already determined by a single 0 h hs-cTnT measurement and thus not diagnostically challenging. When comparing its performance against both the single 0 h hs-cTnT value and the conventional diagnostic thresholds, the results demonstrated that the model possesses good robustness and generalizability to the broader population presenting to the ED, beyond the diagnostically challenging subset originally targeted (Fig S3).

4. **General applicability:**

To verify whether the algorithm can be generalized to all patients presenting to the ED, we additionally evaluated it in a cohort of 380 consecutive ED patients with chest pain, including those who were ruled in after a single 0 h measurement as well as those diagnosed by the 0/1 h algorithm. The results indicated that our model, when integrated with the existing 0/1 h algorithm, can be effectively generalized to real-world clinical practice (Fig 3).

We have revised the manuscript to clarify these issues in the Result section (line 214-224 and line 274-281). Thank you again for your valuable comment.

*Line 214-224*

We firstly designed the study to address an unmet clinical need, patients unable to be definitively ruled in/out for AMI based on their initial (0 h) hs-cTnT values. Accordingly, we focused on individuals whose diagnosis remained uncertain after initial assessment using symptoms, ECG findings, initial hs-cTnT values, and relevant clinical variables such as medical history, medication use, or other cardiovascular diagnostic information, and who therefore underwent continuous monitoring with serial hs-cTnT evaluations before diagnosis (Fig 1A). Following the exclusion of 157 patients diagnosed with STEMI, 1,256 without CBC data [3], 730 without available hs-cTnT within 12 hours before diagnosis, and 26,761 whose diagnosis was already determined by a single 0 h hs-cTnT measurement and thus not diagnostically challenging or within the primary scope of this study, we established ML models with data from 15,096 patients.

*Line 274-281*

Given that one of the strengths of the ML algorithm is its ability to provide risk stratification at 0 hours, we evaluated whether these models could enhance predictive power for patients primarily diagnosed based on the first hs-cTnT measurement (n=26,761, Fig 1A). Nevertheless, when the algorithm was further applied to these excluded patients, it maintained superior predictive performance compared with the 0 h hs-cTnT alone (Fig S3), demonstrating that the model possesses good robustness and generalizability to the broader population presenting to the ED, beyond the diagnostically challenging subset originally targeted.

The routine lab tests were ordered based on the physician’s clinical assessment. This could artificially inflate model performance in the retrospective setting, which should be addressed in the limitation setting.

**Response:**

We sincerely thank the reviewer for this comment. We fully agree with your opinion that routine laboratory testing in the ED often heavily relies on the clinician’s clinical assessment, judgment, and even subjective experience. Such variability can occur not only between individual physicians but also across different healthcare systems, regions, and countries. The presence of numerous missing values in the original dataset indeed reflects this real-world heterogeneity.

We acknowledge that this variability could influence model performance, and we cannot entirely exclude such potential bias in a retrospective study design. To mitigate this issue, we implemented several measures, including enlarging the cohort size, incorporating data collected over a long period, and conducting external validation using an independent medical center dataset.

Nevertheless, we agree that future multinational validations or prospective clinical trials would be essential to definitively address this limitation. We have included these points as part of the discussion on study limitations in the revised manuscript (line 416-421). Thank you very much.

*Line 416-421*

Second, the retrospective nature of this study means that laboratory test ordering in the ED depended on individual physician judgment. This practice-dependent variability led to high proportion of missing values for certain parameters, which might have inadvertently influenced the model’s predictive performance. Future multicenter prospective studies are therefore warranted to confirmed the model’s generalizability and robustness.

Missing values were imputed using the median value stratified by sex. While acceptable for initial analysis, this simple imputation method may fail to capture the complex distribution of clinical data and could introduce bias, especially since some retained features like CKs/PTs had >10% missing values. The authors adequately tested models without these features, but a more sophisticated imputation method (e.g., Multiple Imputation) should be considered.

**Response:**

We sincerely appreciate the reviewer’s insightful comment regarding the imputation strategy. We agree that more sophisticated approaches, such as multiple imputation, could better capture the complex distributions of clinical data and further reduce potential bias. Nevertheless, considering the retrospective, real-world nature of our dataset and the heterogeneous pattern of laboratory test ordering in the ED, we adopted a transparent and reproducible median imputation method to minimize the risk of model overfitting.

Importantly, the imputation was stratified by sex, based on well-established biological and epidemiological evidence that sex is a key determinant of both baseline laboratory distributions and AMI risk. Numerous studies have demonstrated significant sex-related differences in biomarkers relevant to AMI diagnosis, such as cardiac troponin, creatine kinase, and coagulation profiles, which are partly attributable to hormonal influences, muscle mass differences, and cardiovascular physiology [4, 5]. Therefore, imputing missing laboratory values separately for males and females can better preserve the physiological variability inherent to each subgroup and reduce systematic bias.

Moreover, our sensitivity analyses showed that excluding certain variables with >10% missingness, most notably CKs and PTs, which were also associated with the outcome by chi-square test, still led to a statistically significant in predictive performance, indicating that our main conclusions are not driven by the imputation of CK/PT. These results indicate that, although CKs and PTs had relatively high missingness, they nevertheless contributed meaningful discriminative information to the model. We have now added clarification and explicitly acknowledged this limitation in the revised manuscript (line 421-425). Future prospective or multicenter studies could fundamentally overcome the issue of missing values, as laboratory tests and data collection protocols would be pre-specified and standardized prior to patient enrollment. Thank you very much.

*Line 421-425*

Third, while we employed a transparent and practical sex-stratified median imputation approach, we acknowledge that this method may not fully account for complex variable interactions. Adopting more sophisticated techniques, such as multiple or model-based imputation, in future studies could further improve data integrity and model performance.

In my opinion important confounders of NSTEMI diagnoses are missing in the dataset: especially, heart failure and atrial fibrillation. The letter could explain the negative contribution of PT INR in the SHAP analysis.

**Response:**

We sincerely appreciate the reviewer’s insightful and clinically relevant comment. We fully agree that atrial fibrillation and heart failure are important cardiovascular comorbidities that could act as confounders in the diagnosis of STEMI. Both conditions share overlapping clinical presentations and biochemical abnormalities that may complicate the diagnostic process. Indeed, substantial evidence indicates a strong bidirectional relationship between atrial fibrillation and heart failure, where heart failure increases the risk of atrial fibrillation by approximately 44%, and atrial fibrillation increases the risk of heart failure about 18% [6]. These interactions underscore the importance of considering such comorbidities when interpreting cardiac biomarker results and clinical outcomes.

In addition, atrial fibrillation and heart failure are often managed with anticoagulant therapy, which directly influences prothrombin time (PT) and international normalized ratio (INR) levels. Poor INR control has been shown to increase the incidence of myocardial infraction and other adverse cardiovascular events in patients with atrial fibrillation receiving warfarin therapy [7]. Moreover, prolonged PT or elevated INR has been independently associated with long-term mortality in patients with coronary artery disease and AMI [8].

These findings provide a plausible pathophysiological explanation for the negative SHAP contribution of PT/INR observed in our model. Specifically, higher PT/INR levels may reflect chronic anticoagulation therapy, hepatic dysfunction, or non-ischemic cardiac stress rather than type 1 MI, leading to an inverse relationship with NSTEMI diagnosis. We have added this discussion and corresponding references to the revised manuscript (line 373-378) to clarity this interpretation and address the reviewer’s valuable comment.

*Line 373-378*

For instance, atrial fibrillation (AF) and heart failure (HF) are important confounding factors in NSTEMI diagnosis, as both share overlapping symptoms and affect cardiac biomarkers. Patients with AF or HF often receive anticoagulant therapy, resulting in elevated PT/INR levels. This may explain the negative SHAP contribution of PT/INR, since higher values could reflect anticoagulation or non-ischemic cardiac stress rather acute type 1 MI [6-8].

Current challenges in the diagnostic triage of suspected MI are not addressed: Especially, the differentiation of type I and type II MI.

**Response:**

We appreciate the reviewer’s insightful comment regarding this clinically important issue. We agree that differentiating type I MI remains a major challenge in current diagnostic practice. Current consensus definitions (Fourth Universal Definition of MI) and guideline documents make a clear distinction between type 1 and type 2 MI because management strategies differ substantially [9].

In frontline emergency triage, the immediate clinical priority is rapid identification of patients with probable acute ischemia who require urgent reperfusion or early invasive assessment (i.e., those likely to have type 1 MI). Distinguishing type 1 from type 2 MI often requires additional clinical context, hemodynamic data, imaging, and adjudication that are not always available at the time of first blood draw. Consequently, early-rule-in/rule-out tools, including our laboratory-based ML model, are primarily intended to assist raid triage and decision-making rather than to definitively subtype MI [10].

Although this differentiation was not within the primary scope of our study, we agree this is an important limitation. We have added text in the revised manuscript acknowledging these and future work should incorporate adjudicated MI subtype labels and richer hemodynamic/ECG/clinical data to enable subtype-specific models and to assess differential performance (line 425-430). Thank you very much.

*Line 425-430*

Fourth, the model did not distinguish between Type 1 and Type 2 myocardial infarction, which are clinically relevant due to their distinct management strategies. Accurate subtype classification often requires additional clinical and imaging information not immediately available upon presentation. [9] Our model focused on early rule-in/rule-out decisions in the emergency setting; subtype-specific evaluation will be the focus of future research using adjudicated cohorts.

**Reviewer #2:**

Summary:

The paper addresses a clinically relevant problem, which is that the timely diagnosis of NSTEMI is challenging, as current protocols rely on serial hs-cTn tests that may delay clinical decisions and overcrowd emergency departments. In this retrospective study, ML models (XGBoost, Random Forest and Logistic Regression) were experimented with using demographics and routine lab tests to develop a predictive model and hence enable early NSTEMI risk stratification. The final ML model consistently outperformed hs-cTn alone in both internal and external validation sets in terms of AUROC. At clinically relevant thresholds, it achieved a negative predictive value (NPV) of 98.8% (98.5–99.1%) for rule-out and a positive predictive value (PPV) of 78.1% (73.2–82.4%) for rule-in, encompassing 48.3% and 2.6% of patients, respectively. When combined with the established 0 h/1 h algorithm, the ML model further enhanced early decision-making, safely ruling in/out 85.3% of patients within 1 hour, with PPV and NPV reaching 84.9% (79.5–87.7%) and 100% (99.6–100%), respectively. These findings highlight the potential of ML-enhanced triage to accelerate diagnosis and reduce unnecessary observation in the emergency department.

Strengths:
1. Clinical Relevance and Innovation: The study focuses on patients who could not be definitively ruled in or out at presentation, highlighting a clinically challenging subgroup and demonstrating the practical utility and innovation of the proposed diagnostic approach.

**Response:**

Thank you to the reviewer for your recognition and encouragement.

2. Robust Design and Generalizability: The inclusion of both internal and independent external cohorts enhances the study’s reliability and generalizability. While some fine-tuning may be required for the external data, the consistent performance gains achieved through machine learning integration are encouraging.

**Response:**

Thank you very much for your recognition and encouragement. Indeed, given the retrospective nature of our study design and the practical challenges of conducting a prospective trial in the emergency department setting, our study has certain limitations. We have integrated and explicitly stated these limitations in the revised manuscript. Furthermore, considering the importance of generalizability in clinical practice, we additionally developed a risk score model and attempted to integrate it with the existing 0/1 h algorithm to enhance its practical applicability (Fig 3), which yielded highly promising results. We sincerely appreciate your insightful comments.

3. Enhanced Diagnostic Performance: Combining the machine learning model with the established 0 h/1 h algorithm improved diagnostic accuracy and enabled faster patient triage.

**Response:**

Thank you to the reviewer for your recognition and encouragement.

4. Presentation: The study is clearly written, supported by effective figures, with the graphical abstract clearly illustrating the diagnostic process and key outcomes.

**Response:**

Thank you to the reviewer for your recognition and encouragement.

Weaknesses:
1. It is unclear on which validation cohorts the combined ML + 0 h/1 h algorithm was evaluated: both internal and external, or only a subset? If it were conducted only on an internal set, extending the validation to an external set can provide a meaningful assessment of the discussed framework.

**Response:**

Thank the reviewer for pointing out this area that was not clearly described, and we sincerely apologize for the oversight. In the validation of the combined ML + 0/1 h algorithm (Fig 3), all data were derived from the external validation set of the Hsin-Chu Branch of NTUH. The internal training and testing dataset was specifically curated to include patients with multiple hs-cTnT measurements, in order to align with the study hypothesis and address the unmet clinical need; therefore, it was not appropriate for validating the applicability of the ML + 0/1 h algorithm. We have added clarifications regarding the source of the cohort in the revised manuscript (line 138-143 and line 297-299). Please kindly review.

*Line 138-143*

For validation of the model’s clinical applicability and its comparison to the 0 h/1 h algorithm, 380 patients with symptoms of myocardial ischemia were identified from the independent external validation cohort. All patients with available hs-cTnT measurements (a time between samples of >0.5 h to ≤1.5 h) were included to evaluate whether combining the ML model with the 0/1 h algorithm could enhance diagnostic accuracy.

*Line 297-299*

Totally 380 patients with symptoms of myocardial ischemia from the independent external validation cohort and with a time between serial samples of >0.5 h to ≤1.5 h were included to evaluate performances of the 0 h/1 h algorithm (Fig 3A).

2. The study lacks a thorough discussion of its limitations. Two areas, in particular, warrant further attention. First, the absence of deep learning approaches is a notable gap, as such models have consistently demonstrated superior capability in capturing complex ECG patterns and may offer enhanced diagnostic performance [1, 2]. Second, the study does not sufficiently address how inter-subject variability in ECG signals was managed, which is especially important in large and diverse population studies [3]. Beyond, inter-subject variability, assessing the model across subgroups (for example, age groups, genders and ethnicities) can help understand the model’s strengths and limitations along with demonstrating the framework’s applicability and fairness. A detailed discussion of these aspects would strengthen the study’s transparency and contextualize its findings within the broader methodological landscape.
[1] Hannun et al., 2019. Cardiologist-level arrhythmia detection and classification in ambulatory electrocardiograms using a deep neural network.
[2] Devkota et al., 2025. AI analysis for ejection fraction estimation from 12-lead ECG.
[3] Gyawali et al., 200. Learning to disentangle inter-subject anatomical variations in electrocardiographic data.

**Response:**

Thank you very much for insightful and challenging comments. We fully agree that our study still had certain limitations, and we sincerely apologize for not addressing them more comprehensively and explicitly in the Discussion section. Below, we respond to the reviewer’s points in datal and have incorporated corresponding revisions into the revised manuscript. We hope these additions address your concerns and that you understand the current practical constraints we faced.

1. **Regarding deep learning and ECG pattern analysis:**

We completely agree with the reviewer that dep learning approaches applied to ECG pattern recognition can substantially enhance diagnostic performance. Indeed, this highlights an important unmet need and area for improvement in the current clinical workflow for AMI diagnosis and patient triage (rule-in or rule-out) in the ED. Among these diagnostic modalities, ECG analysis is undoubtedly a crucial component. In our study, we chose to focus on improving diagnostic accuracy through routinely available laboratory test data. We believe that the development of new strategies should not aim to replace existing diagnostic frameworks but rather to integrate and complement them – leveraging the strengths of different modalities while addressing their respective limitations. Therefore, we consider it highly promising to integrate our proposed algorithm with deep learning-based ECG pattern classification models in the future to further enhance AMI diagnostic performance, as conceptually illustrated in Fig 3. We have added a statement reflecting this perspective in the revised Discussion section.

2. **Regarding inter-subject variability and subgroup analysis:**

We acknowledge that our study did not explicitly address inter-subject variability in ECG signals, as the primary focus was on the use of objective laboratory data to support AMI diagnosis based on established clinical adjudication (STEMI or NSTEMI). We recognize that differences in ECG interpretation may raise due to variations in physician experience and subjective judgment. To minimize such variability, we included a large dataset spanning multiple years and further validated the model using an independent external cohort from a different population and healthcare system. Nevertheless, we concur with the reviewer that inter-population differences, such as racial, ethnic, or subgroup variations, may influence both AMI prevalence and laboratory test profiles. This underscores the need for larger-scale, potentially multinational validation studies in the future. As for basic demographic characteristics (e.g., age and sex), their relative impact on model prediction can be observed from the SHAP value results (Fig 2B). We have revised the Discussion to include these important considerations.

We deeply grateful for your constructive and well-articulated suggestions. In response, we have added a detailed paragraph in the revised manuscript discussing these limitations, their clinical implications, and future directions (line 398-403). Thank you once again for your valuable input and understanding.

*Line 398-403*

Recent studies have demonstrated that deep learning applied to ECG waveform analysis can markedly enhance AMI diagnosis in emergency care. [11, 12] While such models effectively capture complex temporal and spatial ECG patterns, our approach complements them by focusing on objective biochemical data that are universally available at presentation. We envision that combining our laboratory-based algorithm with ECG-based deep learning systems could further improve diagnostic accuracy and patient triage in future implementations.

Questions:
1. What is the reason behind using only the data from the first admission in case a patient was admitted to the hospital multiple times? Is there any significant reason?

**Response:**

We thank the reviewer for raising this thoughtful question. We included only the first admission for each patient to ensure that the model was trained on encounters representing the initial presentation of acute chest pain, which is the clinically relevant context for early AMI triage in the ED. Subsequent admissions during the 5-years study period may reflect follow-up care, recurrent ischemic events, or presentations driven by other comorbidities, and thus may involve different laboratory testing patterns or clinical decisions processes.

In addition, including multiple admissions from the same individual would disproportionately increase that patient’s influence on model training. Patients with recurrent symptoms often return multiple times within a short window, and counting all visits would artificially inflate their weight in the dataset, potentially biasing the model toward patterns associated with recurrent presentations rather than de novo AMI evaluations. Supporting this concept, prior studies have reported that approximately 27% of AMI survivors revisit the ED for chest pain within one year, and repeat-visit characteristics frequently diverge from first presentations [13].

Importantly, we examined the laboratory data of patients with multiple admissions and found that testing patterns were largely consistent with those of their initial visit, suggesting minimal impact on overall model performance even if subsequent admissions were included. Nonetheless, restricting the dataset to first admissions avoids the above sources of confounding and aligns with real-world clinical workflows, where early diagnostic decision support is intended for initial ED presentations rather than repeat encounters. To clarify this rationale, we have added an explanatory note in the Methods section (line 157-163) of revised manuscript. We appreciate the reviewer’s helpful suggestion and thank you for your understanding.

*Line 157-163*

If a patient was admitted to the hospital multiple times during the 5-year study period, only the data from the first admission were used, as subsequent visits often represent follow-up or unrelated comorbid conditions. This ensured that the model targeted the initial emergency presentation of chest pain and reduced confounding from post-event or chronic processes. In addition, limiting each patient to a single encounter prevented over-weighting individuals with recurrent visits during model training.

2. Why was a random split chosen to split the data into train and test sets? Could there be other criteria?

**Response:**

We appreciate the reviewer’s comment. In this study, we used a random split with stratification by NSTEMI status to perform stratified 10-fold cross-validation because our primary goal was to obtain an unbiased estimate of model performance under the assumption that all observations were drawn from the same underlying population. A random, stratified split is a widely adopted internal validation strategy in predictive modeling [14-16], as it (i) preserves the proportion of NSTEMI and non-NSTEMI cases in each fold, (ii) allows all patients to serve both in training and testing across different folds, and (iii) reduces the dependence on a single arbitrary split by averaging performance over multiple folds.

We agree with the reviewer that other splitting criteria could in principle be considered, such as temporal splits (e.g., training on earlier admissions and testing on later admissions) or site-based splits (e.g., training on some hospitals and testing on another hospital), which may better mimic future deployment or assess transportability across centers. In our dataset, however, there were no major changes in clinical workflow, assay methods, or inclusion criteria over the study period, and the number of cases per site/time period was limited. Therefore, a purely temporal or site-based split would have resulted in very small and unstable test sets. For these reasons, we chose a random, stratified split as a pragmatic and statistically efficient internal validation strategy.

More importantly, to address the concern about generalizability beyond a random internal split, we evaluated the final model in an independent cohort from a different medical center, which serves as an external validation dataset. The consistent performance between the internal cross-validation and this external cohort supports the robustness of our findings. This two-step validation strategy mitigates concerns regarding data leakage or overly optimistic results.[17] Thank you for your understanding.

3. What is the reason for performing missing data imputation based on gender?

**Response:**

We appreciate the reviewer’s thoughtful question. The decision to perform sex-stratified imputation was based on extensive biological and epidemiological evidence demonstrating that sex is a key determinant of both baseline laboratory values and the clinical presentation of AMI. Numerous studies have shown sex-related difference in key biomarkers, including cardiac troponin, creatine kinase, and coagulation parameters, driven by hormonal influences, muscle mass, and cardiovascular physiology [4, 5]. Importantly, the Lancet commission on the global burden of ischemic heart disease also emphasized that woman and men differ in pathophysiological mechanisms, biomarker responses, and diagnostic thresholds for MI [18].

Therefore, imputing missing laboratory values separately for males and females helps preserve the physiological variability inherent to each subgroup and reduces systematic bias introduced by pooling data across sexes. This approach ensures that the imputed distributions more accurately reflect biological reality and improves model fairness and calibration across sex strata. According to the reviewer’s consideration, we added some sentences to clarify this issue in the revised manuscript (line 182-186). Thank you for your understanding.

*Line 182-186*

Missing values of the dataset were imputed by taking the median value of the available non-missing value from the training set after stratification by sex. This sex-stratified approach reflected established biological differences between males and females in laboratory profiles, enhancing model calibration and reducing bias. [18]

4. Thresholds of 1.8 and 38.5 were applied to define low-risk and high-risk groups. Beyond achieving target sensitivity, specificity, PPV, and NPV, do these thresholds have any additional clinical or statistical justification?

**Response:**

We sincerely thank the reviewer for this important issue. The thresholds underlying our low- and high-risk groups were chosen to reflect well-established clinical safety and utility goals for early rule-out and rule-in strategies in the ED.

In clinical practice, early rule-out algorithms for suspected AMI prioritize patient safety by targeting an extremely low miss rate, which has been operationalized in prior studies as either sensitivity ≥99.0% or a NPV ≥99.0-99.5% [19, 20]. These thresholds are consistent with current guideline-endorsed high-sensitivity troponin pathways, including the ESC 0/1 h algorithm, where NPVs above 99.0% are considered acceptable for safe early discharge [21].

Conversely, rule-in thresholds are generally optimized for clinical actionability-balancing false positives and ensuring that patients triaged as high risk truly warrant urgent invasive or therapeutic intervention. Several prior studies and validation framework (e.g., the HEART score and high-sensitivity troponin-based ML models) have adopted PPV around 70.0-80.0% (or specificity ≥90%) as reasonable rule-in targets to preserve clinical utility without excessive overtriage [21, 22].

Based on these principles, we defined thresholds of <1.8 and ≥38.5 in our model to achieve pre-specified operational goal (NPV ~99.5% for rule-out and PPV ~75.0% for rule-in), aligning with the safety and efficiency expectations of real-world ED workflows. We also clarified in the revised manuscript that these thresholds are intended as clinical guided operating points, balancing patient safety and resource optimization, rather than rigid diagnostic cutoffs (line 256-263). Thank you for your understanding.

*Line 256-263*

To achieve an NPV ≥99.5%, a low threshold of 1.8 (95% CI: 1.2–2.4) was used to divide testing sets into low- and not-low-risk groups, where 61.7% (95% CI: 46.7–76.8%) patients were excluded from the diagnosis (Table S5). To reach a PPV ≥75.0%, a high threshold of 38.5 (95% CI: 32.5–44.4) was used to divide the testing set into high- and not-high-risk groups, where 3.0% (95% CI: 2.4–3.6%) patients were ruled in. These thresholds were selected to align with clinical accepted safety and utility benchmarks for early rule-out and rule-in strategies in ED (NPV ~99.0-99.5% for safe discharge and PPV ~70.0-80.0% for actionable diagnosis) [21, 22].

**Reviewer #3:**

Overall:

The authors demonstrate the use of machine learning algorithms to classify NSTEMI patients and non-NSTEMI patients with high AUC on two separate datasets. They clearly demonstrated the utility of their algorithm in the clinical workflow for diagnosing acute myocardial infarction and demonstrated significant generalizability of their model. I think this is a strong manuscript; however, I think a few more comparisons with other established methods in the literature would help contextualize this work. My comments are below:
Comments
• It was not immediately clear why you excluded STEMI patients until I read “For patients exhibiting ST-segment elevation, immediate referral for emergent coronary angiography and revascularization was initiated, following guideline-directed medical therapy” in the supplementary material. I would add this sentence to the methods section when introducing the exclusion criteria.

**Response:**

We apologize for not clearly specifying the exclusion criteria in the initial submission. We fully agree with the reviewer’s suggestion and have now incorporated the statement “For patients exhibiting ST-segment elevation, immediate referral for emergent coronary angiography and revascularization was initiated, following guideline-directed medical therapy”, into the Methods section (line 126-129) to clarify the selection process. We sincerely appreciate your valuable comment and careful review.

*Line 126-129*

Patients with ST-segment elevation myocardial infraction (STEMI) were excluded, as they were promptly referred for emergent revascularization according to guideline-directed therapy; we also excluded cases without available hematological test results or serial hs-cTnT measurement results within 12 hours before diagnosis.

• Were the 14,406 controls people that were not diagnosed with an AMI? This is a bit unclear in the manuscript, please clarify.

**Response:**

We thank the reviewer for this reminder and apologize for not having described this point clearly in the original version. The term “14,406 eligible controls” in Fig 1A indeed refers to individuals who were not ultimately diagnosed with AMI, including neither STEMI nor NSTEMI cases. In response to your comment, we have added a clarifying statement in the revised manuscript to explicitly define this term (line 224-226). Thank you very much.

*Line 224-226*

Among them, 690 were diagnosed with NSTEMI and 14,406 were eligible controls, patients not diagnosed with AMI, including neither STEMI nor NSTEMI cases (Table 1)..

• For the feature selection, was the analysis shown in Fig S1 using just the data from the NTUH? Or did it include both datasets? Ideally it was done just on the first dataset to avoid data leakage.

**Response:**

We thank the reviewer for this helpful reminder, and we apologize for not having clearly stated this point in the original manuscript. For the feature selection process, we indeed used data exclusively from NTUH. This approach was intentionally adopted to prevent potential data leakage. Additionally, given that laboratory testing methods and data distributions may vary across different healthcare systems, it would not be appropriate to combine datasets for feature selection.

We appreciate the reviewer insightful comment and have added clarifying statements in the revised manuscript to avoid any misunderstanding (line 169-171).

*Line 169-171*

Feature selection was performed using data exclusively from NTUH to avoid potential data leakage, without incorporating data from the external validation cohort, and the resulting features were subsequently applied to model training and validation.

• You mention in the discussion that you wanted to avoid the use of neural networks to maintain simplicity and interpretability. However, it would be interesting to baseline your method against a more complex algorithm to check that you are not sacrificing a significant amount of model performance for interpretability. SHAP analysis can also be used for neural network models to uncover feature importance as well.

**Response:**

We sincerely appreciate the reviewer’s comment and fully agree that model interpretability should not come at expense of diagnostic performance. Our original description may lead to misunderstanding. Indeed, neural network based architectures, when properly trained and interpreted (e.g., using SHAP or LIME), can provide powerful nonlinear modeling capacity for complex clinical data.

In response to the reviewer’s suggestion, we evaluated a neural network model using a multilayer perceptron (MLP) architecture, which achieved an AUROC of 0.877 (95% CI: 0.867–0.888) and an APPRC of 0.511 (95% CI: 0.481–0.540) for the testing set (revised Fig S4). While these results were slightly inferior to those obtained with the XGBoost and RF models, our decision to adopt XGBoost was not based on a rejection of deep learning approaches, but rather on a trade-off between performance, stability, and clinical interpretability.

As supported by prior literature, for structured and tabular datasets such as routine laboratory data, tree-based ensemble methods like XGBoost often achieve performance comparable to, or even exceeding, that of deep neural networks, while retaining greater transparency and computational efficiency. [23, 24] Moreover, in emergency medicine settings, where rapid decision-making and explainability are critical for clinical acceptance, the ability to provide feature-level reasoning confers an important practical advantage [25].

We fully acknowledge and share the reviewer’s perspective hat more complex models, including neural networks with SHAP-based interpretability, may further enhance diagnostic performance in future studies. To avoid potential misunderstanding and address the reviewer’s comment, we have added a Fig S4 and revised certain statements in the revised manuscript (line 321-334).

*Revised Fig S4*

**Fig S4. Prediction performance of the neural network model.** Comparison of the area under receiver-operating-curves (AUROC) (upper left) and average precision of precision-recall curves (APPRC) (upper right) generated from the testing set for the XGBoost (XGB) model, random forest (RF) model, and multilayer perceptron (MLP) architecture. Solid lines represent medians, and color zones represent 95% CI.

*Line 321-334*

Although numerous statistical and neural network models have been proposed for diagnosing and prognosing acute cardiovascular conditions, including AMI [26-28], their clinical adoption has been limited by model complexity, substantial number of required variables insufficient validation [29]. In parallel, a neural network model using a multilayer perceptron (MLP) architecture was also evaluated and achieved an AUROC of 0.877 (95% CI: 0.867–0.888) and an APPRC of 0.511 (95% CI: 0.481–0.540) in the testing set (Fig S4). While deep learning architectures can capture higher-order nonlinearities, prior evidence suggests that for structured laboratory datasets, gradient boosting methods such as XGBoost can provide comparable performance while offering greater transparency, interpretability, robustness, and computational efficiency, features essential for clinical adoption in emergency medicine [24]. Therefore, the selection of XGBoost as the primary modeling approach in this study was guided not solely by predictive performance, but by a balanced consideration of diagnostic accuracy, interpretability, and real-word clinical feasibility to support practical deployment.

• Additionally, it would be useful to compare your method to other methods presented in the literature before you like the MI^3 algorithm in your references ([19, 20]). They use a gradient boosting algorithm which was not tried in this work.

**Response:**

We appreciate the reviewer’s insightful comment and the opportunity to elaborate on the comparison with the MI^3^ algorithm [22]. The MI^3^ model was constructed using age, sex, and paired high-sensitivity cardiac troponin I measurements within a gradient boosting framework, achieving an NPV of 99.7%, sensitivity of 97.8%, PPV of 71.8%, and specificity of 96.7%. Our model demonstrated comparable performance (NPV 98.8%, sensitivity 91.3%, PPV 74.3%, specificity 99.2%) and even slightly higher PPV and specificity (Table 2).

Importantly, our approach emphasize real-world applicability: prediction can be made from a single 0 h blood draw, without requiring serial cardiac troponin I testing. This design aligns with the clinical workflow in emergency settings, providing earlier decision support and reducing patient waiting time and anxiety. Moreover, when integrated with the ESC 0/1 h algorithm, our model achieved superior performance (NPV, sensitivity, PPV, and specificity of 100%, 100%, 84.9%, and 96.5%, respectively) (Fig 3).

Regarding the reviewer’s comment on the use of gradient boosting, we had tried gradient boosting model where the AUROC is 0.921 (95% CI: 0.908-0.933) and APPRC is 0.600 (95% CI: 0.546-0.654) (data not showed) for the testing set, which showed inferior performances to the XGB model (data in below). We would like to clarify that our study employed Extreme Gradient Boosting (XGBoost), an advanced implementation of gradient boosting that incorporates regularization, parallel computation, and optimized tree learning to reduce overfitting and improve computational efficiency [23]. Numerous comparative studies have shown that XGBoost consistently outperforms conventional gradient boosting algorithms across various biomedical applications [25, 30]. Therefore, we believe our decision of XGBoost is methodologically sound and well suited for the current dataset.

Finally, we would like to clarify that our intent is not to position our model as a direct replacement for existing approaches such as the MI^3^ model, but rather to complement them within an integrated diagnostic framework. While we have conducted a formal comparison with the MI^3^ gradient boosting approach and observed favorable performance of our XGBoost model, we believe that emphasizing relative superiority may detract from the broader clinical message of collaborative algorithm integration. Instead, consistent with current trends toward hybrid and pragmatic diagnostic strategies, our model is designed to function alongside established tools such as the ESC 0/1 h pathway, MI^3^, or ECG-based AI systems to support more efficient and adaptable AMI triage. Therefore, we have chosen to present the detailed comparative results in this response letter, while maintaining a balanced and clinically oriented discussion in the revised manuscript (line 404-412). We sincerely thank the reviewer for this valuable suggestion and for your understanding.

*Data by gradient boosting (GBDT)*

**Fig. Prediction performance of the gradient boosting model.** Comparison of the area under receiver-operating-curves (AUROC) (upper left) and average precision of precision-recall curves (APPRC) (upper right) generated from the testing set by the XGBoost (XGB) model, random forest (RF) model, and gradient boosting decision tree (GBDT). Solid lines represent medians, and color zones represent 95% CI.

*Line 404-412*

Similarly, the MI^3^ model proposed by Than et al. employed age, sex, and paired hs-cTnI values through a gradient boosting framework to predict AMI, achieving an NPV of 99.7% and a PPV of 78.1. [22] Our results were comparable or slightly superior in PPV and specificity, while requiring only a single 0 h blood draw. Moreover, by employing XGBoost, an advanced and regularized form of gradient boosting, our model achieved enhanced robustness and interpretability. Importantly, rather than serving as a methodological replacement, our algorithm was designed to complement established strategies such as the ESC 0/1 pathway or existing AI tools, thereby supporting a more efficient and clinically adaptable diagnostic workflow.

**References**

1.	Gandhi S, Garratt KN, Li S, Wang TY, Bhatt DL, Davis LL, et al. Ten-Year Trends in Patient Characteristics, Treatments, and Outcomes in Myocardial Infarction From National Cardiovascular Data Registry Chest Pain-MI Registry. Circ Cardiovasc Qual Outcomes. 2022;15(1):e008112. Epub 2022/01/19. doi: 10.1161/CIRCOUTCOMES.121.008112.

2.	Greenberg SA, Cohen N, Shopen N, Mordechai RA, Zeltser D, Werthein J. Outcomes of ED chest pain visits: the prognostic value of negative but measurable high-sensitivity cardiac troponin (hs-cTn) levels. BMC Emerg Med. 2024;24(1):223. Epub 2024/11/27. doi: 10.1186/s12873-024-01128-1.

3.	Gould MK, Huang BZ, Tammemagi MC, Kinar Y, Shiff R. Machine Learning for Early Lung Cancer Identification Using Routine Clinical and Laboratory Data. Am J Respir Crit Care Med. 2021;204(4):445-53. Epub 2021/04/07. doi: 10.1164/rccm.202007-2791OC.

4.	Chapman AR, Adamson PD, Shah ASV, Anand A, Strachan FE, Ferry AV, et al. High-Sensitivity Cardiac Troponin and the Universal Definition of Myocardial Infarction. Circulation. 2020;141(3):161-71. Epub 2019/10/08. doi: 10.1161/CIRCULATIONAHA.119.042960.

5.	Twerenbold R, Jaeger C, Rubini Gimenez M, Wildi K, Reichlin T, Nestelberger T, et al. Impact of high-sensitivity cardiac troponin on use of coronary angiography, cardiac stress testing, and time to discharge in suspected acute myocardial infarction. Eur Heart J. 2016;37(44):3324-32. Epub 2016/07/01. doi: 10.1093/eurheartj/ehw232.

6.	Zhang Z, Li L, Hu Z, Zhou L, Zhang Z, Xiong Y, et al. Causal effects between atrial fibrillation and heart failure: evidence from a bidirectional Mendelian randomization study. BMC Med Genomics. 2023;16(1):187. Epub 2023/08/15. doi: 10.1186/s12920-023-01606-8.

7.	Raatikainen MJP, Penttila T, Korhonen P, Mehtala J, Lassila R, Lehto M. The quality of warfarin therapy and CHA2DS2-VASc score associate with the incidence of myocardial infarction and cardiovascular outcome in patients with atrial fibrillation: data from the nationwide FinWAF Registry. Eur Heart J Cardiovasc Pharmacother. 2018;4(4):211-9. Epub 2018/03/08. doi: 10.1093/ehjcvp/pvy009.

8.	Liu L, Ying M, Chen S, Li Q, Chen G, Li H, et al. The association between prothrombin time-international normalized ratio and long-term mortality in patients with coronary artery disease: a large cohort retrospective study with 44,662 patients. BMC Cardiovasc Disord. 2022;22(1):297. Epub 2022/06/30. doi: 10.1186/s12872-022-02619-4.

9.	Thygesen K, Alpert JS, Jaffe AS, Chaitman BR, Bax JJ, Morrow DA, et al. Fourth Universal Definition of Myocardial Infarction (2018). Circulation. 2018;138(20):e618-e51. Epub 2018/12/21. doi: 10.1161/CIR.0000000000000617.

10.	Bergmark BA, Mathenge N, Merlini PA, Lawrence-Wright MB, Giugliano RP. Acute coronary syndromes. Lancet. 2022;399(10332):1347-58. Epub 2022/04/04. doi: 10.1016/S0140-6736(21)02391-6.

11.	Gyawali PK, Murkute JV, Toloubidokhti M, Jiang X, Horacek BM, Sapp JL, et al. Learning to Disentangle Inter-Subject Anatomical Variations in Electrocardiographic Data. IEEE Trans Biomed Eng. 2022;69(2):860-70. Epub 2021/08/31. doi: 10.1109/TBME.2021.3108164.

12.	Hannun AY, Rajpurkar P, Haghpanahi M, Tison GH, Bourn C, Turakhia MP, et al. Cardiologist-level arrhythmia detection and classification in ambulatory electrocardiograms using a deep neural network. Nat Med. 2019;25(1):65-9. Epub 2019/01/09. doi: 10.1038/s41591-018-0268-3.

13.	Holzmann MJ, Andersson T, Doemland ML, Roux S. Recurrent myocardial infarction and emergency department visits: a retrospective study on the Stockholm Area Chest Pain Cohort. Open Heart. 2023;10(1). Epub 2023/06/30. doi: 10.1136/openhrt-2022-002206.

14.	Alam MA, Sajib M, Rahman F, Ether S, Hanson M, Sayeed A, et al. Implications of Big Data Analytics, AI, Machine Learning, and Deep Learning in the Health Care System of Bangladesh: Scoping Review. J Med Internet Res. 2024;26:e54710. Epub 2024/10/28. doi: 10.2196/54710.

15.	Beam AL, Kohane IS. Big Data and Machine Learning in Health Care. JAMA. 2018;319(13):1317-8. Epub 2018/03/14. doi: 10.1001/jama.2017.18391.

16.	Ngiam KY, Khor IW. Big data and machine learning algorithms for health-care delivery. Lancet Oncol. 2019;20(5):e262-e73. Epub 2019/05/03. doi: 10.1016/S1470-2045(19)30149-4.

17.	Riley RD, Ensor J, Snell KI, Debray TP, Altman DG, Moons KG, et al. External validation of clinical prediction models using big datasets from e-health records or IPD meta-analysis: opportunities and challenges. BMJ. 2016;353:i3140. Epub 2016/06/24. doi: 10.1136/bmj.i3140.

18.	Wenzl FA, Kraler S, Ambler G, Weston C, Herzog SA, Raber L, et al. Sex-specific evaluation and redevelopment of the GRACE score in non-ST-segment elevation acute coronary syndromes in populations from the UK and Switzerland: a multinational analysis with external cohort validation. Lancet. 2022;400(10354):744-56. Epub 2022/09/02. doi: 10.1016/S0140-6736(22)01483-0.

19.	Kavsak PA, Neumann JT, Cullen L, Than M, Shortt C, Greenslade JH, et al. Clinical chemistry score versus high-sensitivity cardiac troponin I and T tests alone to identify patients at low or high risk for myocardial infarction or death at presentation to the emergency department. CMAJ. 2018;190(33):E974-E84. Epub 2018/08/22. doi: 10.1503/cmaj.180144.

20.	Pickering JW, Greenslade JH, Cullen L, Flaws D, Parsonage W, Aldous S, et al. Assessment of the European Society of Cardiology 0-Hour/1-Hour Algorithm to Rule-Out and Rule-In Acute Myocardial Infarction. Circulation. 2016;134(20):1532-41. Epub 2016/10/19. doi: 10.1161/CIRCULATIONAHA.116.022677.

21.	Collet JP, Thiele H, Barbato E, Barthelemy O, Bauersachs J, Bhatt DL, et al. 2020 ESC Guidelines for the management of acute coronary syndromes in patients presenting without persistent ST-segment elevation. Eur Heart J. 2021;42(14):1289-367. Epub 2020/08/30. doi: 10.1093/eurheartj/ehaa575.

22.	Than MP, Pickering JW, Sandoval Y, Shah ASV, Tsanas A, Apple FS, et al. Machine Learning to Predict the Likelihood of Acute Myocardial Infarction. Circulation. 2019;140(11):899-909. Epub 2019/08/17. doi: 10.1161/CIRCULATIONAHA.119.041980.

23.	Chen T, Guestrin C. XGBoost: A Scalable Tree Boosting System. 2016. p. 785-94.

24.	Shwartz-Ziv R, Armon A. Tabular data: Deep learning is not all you need. Information Fusion. 2022;81:84-90. doi: <https://doi.org/10.1016/j.inffus.2021.11.011>.

25.	Rajkomar A, Dean J, Kohane I. Machine Learning in Medicine. N Engl J Med. 2019;380(14):1347-58. Epub 2019/04/04. doi: 10.1056/NEJMra1814259.

26.	Eggers KM, Ellenius J, Dellborg M, Groth T, Oldgren J, Swahn E, et al. Artificial neural network algorithms for early diagnosis of acute myocardial infarction and prediction of infarct size in chest pain patients. Int J Cardiol. 2007;114(3):366-74. Epub 2006/06/27. doi: 10.1016/j.ijcard.2005.12.019.

27.	Green M, Björk J, Forberg J, Ekelund U, Edenbrandt L, Ohlsson M. Comparison between neural networks and multiple logistic regression to predict acute coronary syndrome in the emergency room. Artif Intell Med. 2006;38(3):305-18. Epub 2006/09/12. doi: 10.1016/j.artmed.2006.07.006.

28.	Wessler BS, Lai Yh L, Kramer W, Cangelosi M, Raman G, Lutz JS, et al. Clinical Prediction Models for Cardiovascular Disease: Tufts Predictive Analytics and Comparative Effectiveness Clinical Prediction Model Database. Circ Cardiovasc Qual Outcomes. 2015;8(4):368-75. Epub 2015/07/15. doi: 10.1161/circoutcomes.115.001693.

29.	Siontis GC, Tzoulaki I, Castaldi PJ, Ioannidis JP. External validation of new risk prediction models is infrequent and reveals worse prognostic discrimination. J Clin Epidemiol. 2015;68(1):25-34. Epub 2014/12/03. doi: 10.1016/j.jclinepi.2014.09.007.

30.	Lundberg SM, Erion G, Chen H, DeGrave A, Prutkin JM, Nair B, et al. From Local Explanations to Global Understanding with Explainable AI for Trees. Nat Mach Intell. 2020;2(1):56-67. Epub 2020/07/02. doi: 10.1038/s42256-019-0138-9.
